# Pulmonary Leiomyomatosis in a Postmenopausal Patient After Previous Hysterectomy: A Case Report

**DOI:** 10.1155/crog/9549502

**Published:** 2026-01-29

**Authors:** Ján Varga, Karolina Just, Alexander Ostró

**Affiliations:** ^1^ Department of Gynecology and Obstetrics, Faculty of Medicine, P. J. Šafárik University and L. Pasteur University Hospital, Košice, Slovakia; ^2^ Department of Gynecology, Obstetrics and Gynecological Oncology, Healthcare Center, Świętochłowice, Poland

**Keywords:** leiomyoma, leiomyoma dissemination, metastasising leiomyomatosis, pulmonary leiomyomatosis, uterine leiomyoma

## Abstract

**Objective:**

Uterine leiomyoma represents one of the most common pathologies affecting women of reproductive age, often presenting with lower abdominal pain and abnormal bleeding. Benign metastasising leiomyomatosis (BML) is a rare tumour originating from uterine leiomyomas, with the lungs being a common extrauterine location. The aetiopathogenesis remains unclear, and no standardised treatment protocols exist due to the rarity of the disease.

**Case Report:**

We present a case of a patient who developed pulmonary BML 7 years after hysterectomy for uterine leiomyoma. Despite initial hormonal therapy and various interventions, the patient′s condition progressively worsened, leading to pulmonary hypertension, cardiac decompensation and ultimately death. Despite consultations with oncologists and treatment with aromatase inhibitors and doxorubicin, the disease proved refractory to treatment.

**Conclusion:**

BML remains a challenging condition to manage due to its low incidence and lack of standardised treatment protocols. Multidisciplinary approaches are essential, and further research is needed to establish better treatment guidelines.

## 1. Introduction

Benign metastasising leiomyomatosis (BML) is a rare tumour composed of smooth muscle cells originating from a uterine leiomyoma. Its extrauterine locations include the heart, liver, bones, muscles, pancreas and lungs [1, 2]. The diagnosis is usually made in premenopausal women who previously underwent hysterectomy or myomectomy due to uterine leiomyomas, though it may also be diagnosed in women without previous uterine surgery [3]. In the pulmonary form of BML, cough, dyspnoea and chest pain are often seen [4]. Histologically, BML resembles uterine leiomyoma, with oestrogen and progesterone receptor expression observed [3, 5].

The aetiology of BML remains unclear and is based on various hypotheses. One of them defines the BML as ‘low‐grade’ leiomyosarcoma, whilst another suggests an intravascular dissemination of uterine leiomyoma after its resection during primary surgery. Another theory indicates focal proliferation of smooth muscle cells due to an oestrogen and progesterone stimulation [2].

Given that BML is often asymptomatic, it may present as an incidental finding on CT or MR [1, 4]. In differential diagnosis, the metastases of malignant tumours, sarcoidosis, amyloidosis, tuberculosis and parasitic and fungal infections should be excluded. The final diagnosis is confirmed by histological examination of biopsied tissue.

Due to the disease′s rarity, there are no standardised protocols. In asymptomatic patients, conservative management with regular imaging examination is usually performed. In an active approach, uterine leiomyoma removal is indicated once diagnosed, followed by affecting sex hormone levels by performing bilateral oophorectomy, administration of GnRH analogues, selective oestrogen receptor modulators or aromatase inhibitors. In the postmenopausal period, a decrease in BML lesion size can be seen due to hormone depletion. Another treatment option is a surgical resection of pulmonary lesions [4]. Lung transplantation has not yet been performed in pulmonary BML patients.

## 2. Case Presentation

This case report presents a 55‐year‐old patient with a history of hypertension and autoimmune thyroiditis. In 1991, she underwent diagnostic laparoscopy due to primary infertility and, in 2011 (at 48 years), abdominal hysterectomy due to uterine leiomyoma. At this time, observation only was recommended.

**Figure 1:**
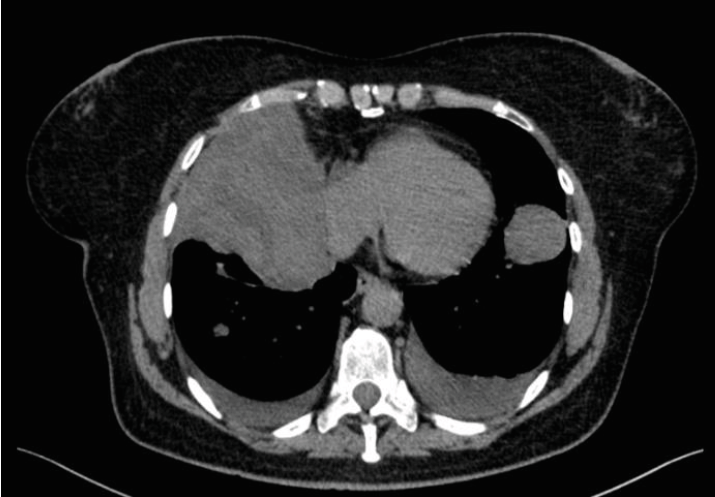
CT picture in the transverse plane. The finding at the time of diagnosis (12/2018).

In December 2018, the patient was hospitalised at the Pulmonology Department due to a dry, irritating cough. X‐ray and CT examinations revealed multiple solid nodules with pleural infiltration (Figure 1). The CT‐guided biopsy was performed. The histologic examination confirmed smooth muscle cells with uncertain biological behaviour, without atypia, without mitotic activity, SMA+, desmin+ and Ki‐67 < 1%—consistent with the diagnosis of pulmonary leiomyomatosis. The oestrogen and progesterone receptors showed diffuse positivity in 99% of tumour cells. The diagnosis was pulmonary BML (Figure 2). There was an elevation of the CA125 level seen (CA125:203.63 U/mL). Transvaginal and transabdominal ultrasound did not reveal any pathology in the pelvis or abdominal cavity. Due to the extent of lesions, the initial finding was defined as unresectable. No oncologic treatment was indicated.

Figure 2Histological pictures. (a) Pulmonary leiomyomatosis (haematoxylin–eosin, original magnification ×400, bar = 50 *μ*m), (b) uterine leiomyomatosis (haematoxylin–eosin, original magnification ×400, bar = 50 *μ*m) and (c) pulmonary lymphangioleiomyomatosis (haematoxylin–eosin, original magnification ×200, bar = 200 *μ*m).

**Figure:**
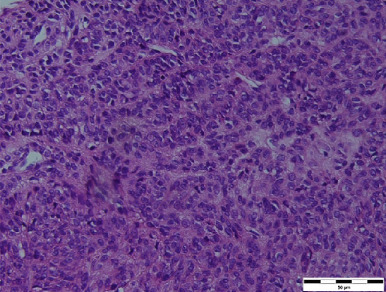
(a)

**Figure:**
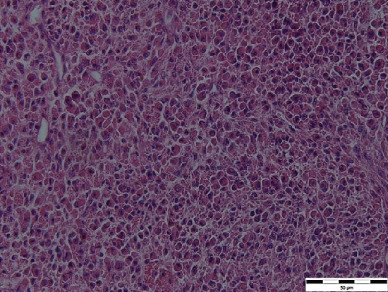
(b)

**Figure:**
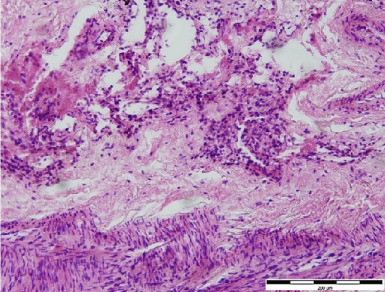
(c)

Hormonal therapy was suggested. Ulipristal acetate was administered from March to May 2019. Whilst the patient remained stable initially, her symptoms progressed in July 2019, and the patient was hospitalised at the Chest Surgery Centre. Bilateral pleural effusion was confirmed, and the decompressive drainage and the left‐sided video‐assisted thoracoscopic surgery were performed. The biopsy confirmed pulmonary BML. Despite the patient′s postmenopausal status, in October 2019, laparoscopic bilateral adnexectomy with peritoneal lavage cytology was performed to suppress residual hormone production, limit disease progression and exclude additional BML deposits in the abdominal cavity. Histological and cytological examinations did not show any pathology. The consultation with the radiation oncologist did not indicate stereotactic radiotherapy due to the extent of lesions.

Slight progression of the disease was noticed in January 2020 during a CT examination. Core cut CT‐guided biopsy excluded malignant transformation of the lesion. In June 2020, due to the dyspnoea accentuation and progression of right‐sided pleural effusion, the drainage was performed. From January 2020, the patient was repeatedly referred to the Transplant Centre. Due to exhaustion of all conservative therapies and due to the diagnosis of tumour metastatic disease, lung transplantation was contraindicated.

**Figure 3:**
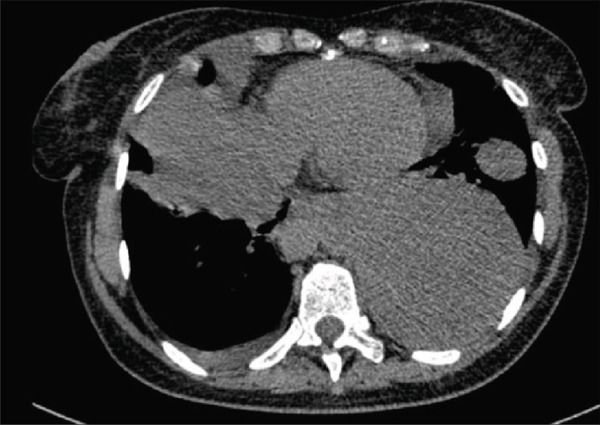
CT picture in the transverse plane. The finding at the time of disease progression (5/2020).

After the consultation with the Department of Oncology at the Centre Léon Bérard in Lyon, which is a reference centre for adult rare solid cancers, our patient started a 2‐month therapy with aromatase inhibitors. After 2 months of treatment, CT confirmed a stationary finding; therefore, the patient continued therapy. The worsening of symptoms and the progression of CT findings were noticed after 4 months, and the treatment with aromatase inhibitors was replaced by doxorubicin every 3 weeks (Figure 3). However, the patient′s condition deteriorated rapidly, dyspnoea progressed and, due to the secondary pulmonary hypertension, right cardiac decompensation developed; the patient died in February 2021.

## 3. Discussion

Uterine leiomyomas are amongst the most common benign tumours encountered in clinical practice. However, the spread to extrauterine locations, such as the lungs, is rare [6]. The diagnosis of BML is made approximately 10 years after surgery for uterine leiomyoma [7]. This is usually a diagnosis of already symptomatic disease, and the presence of microscopic BML can be found even earlier. The share of the ‘low‐grade’ hypothesis on disease development is supported by histological findings in the presented case [8].

A significant number of patients are asymptomatic at the time of diagnosis of pulmonary BML. The presence of symptoms and their severity depend on the number of lesions, localisation and their size [7, 9].

Most cases of pulmonary BML include bilateral dissemination, and only 13% of patients show unilateral disease [10]. More common bilateral infiltration supports the hypothesis of vascular dissemination of the disease. The question of why the lungs are predominantly affected remains unanswered. The explanation may also be the degree of sensitivity of the lung parenchyma to sex hormones in the circulation. The rare incidence of BML compared to extremely frequent surgeries of uterine myomas does not allow to clearly define the risk of BML development after uterine surgery. Moreover, the disease can be seen even in patients without a previous uterine surgery, and the uterine leiomyoma may be diagnosed at the time of BML diagnosis. The reason why some BML patients do not have uterine leiomyomas can be due to small asymptomatic lesions of BML in the lung parenchyma which were missed during diagnosis. The theory of ‘de novo’ formation of pulmonary leiomyomas is less likely [11].

Given the low incidence of BML, standardised treatment guidelines are lacking, so case reports remain necessary to understand the disease. Bilateral oophorectomy is often recommended in postmenopausal patients. For asymptomatic patients, observation with regular imaging seems to be most effective. In symptomatic patients, surgical resection of pulmonary nodules may be necessary. Pharmacological and hormonal treatment is typically reserved for patients with unresectable disease [12, 13]. Lung transplantation, though not yet performed for BML, may be considered in advanced cases. However, further multidisciplinary discussion is needed.

## Consent

Written consent for the publication of their clinical details and image was obtained from the patient. A copy of the consent form is available for review.

## Conflicts of Interest

The authors declare no conflicts of interest.

## Funding

This work was funded by the Kultúrna a Edukacná Grantová Agentúra MŠVVaŠ SR (10.13039/501100006108, 024SPU‐4/2023).

## Supporting information


**Supporting Information** Additional supporting information can be found online in the Supporting Information section.

## Data Availability

The data that support the findings of this study are available from the corresponding author upon reasonable request.
