# Supplementary material for: Pulmonary Leiomyomatosis in a Postmenopausal Patient After Previous Hysterectomy: A Case Report
Source: Case Rep Obstet Gynecol. 2026 Jan 29;2026:9549502. doi: 10.1155/crog/9549502 (PMC12854095; doi:10.1155/crog/9549502)
Supplement: Supplementary file 1 — Supporting Information Additional supporting information can be found online in the Supporting Information section. [file CROG-2026-9549502-s001.pdf]

CARE
case report guidelines

CARE Checklist of information to include when writing a case report

(cc) BY-NC-ND

Topic	Item	Checklist item description	Reported on Line
**Title**	1	The diagnosis or intervention of primary focus followed by the words "case report"	3, 4, p.1
**Key Words**	2	2 to 5 key words that identify diagnoses or interventions in this case report, including "case report"	42, 43, p.2
**Abstract (no references)**	3a	Introduction: What is unique about this case and what does it add to the scientific literature?	27-31, p.2
	3b	Main symptoms and/or important clinical findings	32-35, p.2
	3c	The main diagnoses, therapeutic interventions, and outcomes	32-33, 35-37, p.2
	3d	Conclusion—What is the main "take-away" lesson(s) from this case?	38-40, p.2
**Introduction**	4	One or two paragraphs summarizing why this case is unique (**may include references**)	47-74, p.3
**Patient Information**	5a	De-identified patient specific information.	77-80, p.4
	5b	Primary concerns and symptoms of the patient.	82-84, p.4
	5c	Medical, family, and psycho-social history including relevant genetic information	77-80, p.4
	5d	Relevant past interventions with outcomes	78-79, p.4
**Clinical Findings**	6	Describe significant physical examination (PE) and important clinical findings.	82-83, p.4
**Timeline**	7	Historical and current information from this episode of care organized as a timeline	78-118, pp. 4-5
**Diagnostic Assessment**	8a	Diagnostic testing (such as PE, laboratory testing, imaging, surveys).	83-90, p.4
	8b	Diagnostic challenges (such as access to testing, financial, or cultural)	N/A
	8c	Diagnosis (including other diagnoses considered)	88, p.4
	8d	Prognosis (such as staging in oncology) where applicable	90-91, p.4
**Therapeutic Intervention**	9a	Types of therapeutic intervention (such as pharmacologic, surgical, preventive, self-care)	93-102, 106-107, 111-116, pp. 4-5
	9b	Administration of therapeutic intervention (such as dosage, strength, duration)	93-94, 112-116, pp.4-5
	9c	Changes in therapeutic intervention (with rationale)	111-116, p.5
**Follow-up and Outcomes**	10a	Clinician and patient-assessed outcomes (if available)	116-118, p.5
	10b	Important follow-up diagnostic and other test results	104-107, 113-115, pp.4-5
	10c	Intervention adherence and tolerability (How was this assessed?)	93-118, pp.4-5
	10d	Adverse and unanticipated events	114-118, p.5
**Discussion**	11a	A scientific discussion of the strengths AND limitations associated with this case report	144-145, 150-151, p.6
	11b	Discussion of the relevant medical literature **with references**.	121-142, pp. 5-6
	11c	The scientific rationale for any conclusions (including assessment of possible causes)	132-142, pp. 5-6
	11d	The primary "take-away" lessons of this case report (without references) in a one paragraph conclusion	144-151, p.6
**Patient Perspective**	12	The patient should share their perspective in one to two paragraphs on the treatment(s) they received	N/A
**Informed Consent**	13	Did the patient give informed consent? Please provide if requested	**Yes** ☑ **No** ☐
